# Lobular endocervical glandular hyperplasia diagnosed during surveillance for Peutz–Jeghers Syndrome: A case report

**DOI:** 10.1016/j.gore.2024.101673

**Published:** 2025-01-16

**Authors:** Takayuki Ichinose, Kazuki Takasaki, Yuko Takahashi, Mana Hirano, Haruka Nishida, Haruko Hiraike, Yuko Sasajima, Kazunori Nagasaka

**Affiliations:** a Department of Obstetrics and Gynecology, Teikyo University School of Medicine, Itabashi-ku, Tokyo 173-8605, Japan; b Department of Pathology, Teikyo University School of Medicine, Itabashi-ku, Tokyo 173-8605, Japan

**Keywords:** Peutz–Jeghers syndrome, STK11, Lobular endocervical glandular hyperplasia, STK11IP

## Abstract

**Background:**

Lobular endocervical glandular hyperplasia (LEGH) is a benign cervical condition that has been proposed as a precursor lesion to minimal deviation adenocarcinoma (MDA), a rare but highly aggressive subtype of well-differentiated gastric-type endocervical adenocarcinoma (GAS). MDA is more frequently observed in patients with Peutz–Jeghers syndrome (PJS) and is strongly associated with mutations in the STK11 gene. While LEGH is not inherently linked to PJS, its potential progression to MDA warrants vigilance, particularly in patients with PJS due to their heightened risk of gynecologic malignancies. Here, we report a case of LEGH diagnosed during surveillance for PJS, analyzed via whole genome sequencing. LEGH is a benign cervical condition, considered a precursor lesion to minimal deviation adenocarcinoma, often observed in patients with Peutz–Jeghers syndrome (PJS) and potentially linked to mutations in the *STK11* gene.

**Case:**

A 23-year-old woman, diagnosed with PJS at age 11, was referred to a gynecologist after a cystic lesion was detected in the cervix during a follow-up computed tomography scan. Initial examinations, including imaging and colposcopy, did not indicate LEGH or a malignant tumor. Imaging alone is insufficient to exclude premalignant or malignant conditions, as abnormal cervical lesions often require biopsy for a definitive diagnosis. However, cervical cytology showed nuclear atypia was minimal, but some clusters exhibited disordered alignment, and the cytoplasm contained yellowish mucus suggestive of LEGH. Based on these findings, cytology follow-up was planned. However, the patient did not return for further follow-up. After one year and five months, the patient presented with increased mucous vaginal discharge. Cervical cytology indicated atypical glandular cells, and magnetic resonance imaging with contrast enhancement revealed an enlarged cervical lesion, suggesting minimal deviation adenocarcinoma. We performed cervical conization, and a histopathological examination helped confirm LEGH. High-throughput next-generation sequencing of the excised cervical tissue revealed a missense mutation in the serine/threonine kinase 11 (*STK11*) gene on chromosome 19 (c.1062C > G) and three missense mutations in STK11 interacting protein (*STK11IP*) on chromosome 2 (c.2G > T, c.1687G > A, c.2255C > T). Mutations in STK11, particularly those affecting its regulatory domains, may significantly increase cancer risk in patients with PJS, and that STK11IP plays a crucial role in modulating STK11 activity. The patient, seeking to preserve fertility, has been monitored for five years post-surgery without evidence of malignant transformation. Continuous monitoring with periodic imaging and cytological assessments has shown no evidence of malignant transformation. The absence of elevated tumor markers further supports the conservative approach. While there are no tumor markers specific to cervical cancer, carcinoembryonic antigen (CEA), cancer antigen 125 (CA125), and carbohydrate antigen 19–9 (CA19-9) were measured as part of the screening process in this case.

**Conclusion:**

We conclude that whole genome sequencing in patients with PJS and LEGH could be pivotal in predicting cervical malignancy. This case emphasizes the utility of whole genome sequencing in predicting the risk of cervical malignancy in patients with PJS and LEGH and highlighting the critical role of genetic factors in the management and surveillance of these patients. Continuous monitoring post-surgery showed no evidence of malignant transformation, supporting a conservative approach in fertility-preserving treatment scenarios.

## Highlights

• We report a case of lobular endocervical glandular hyperplasia (LEGH)
• The lesion was detected during surveillance for Peutz–Jeghers syndrome (PJS)
• A missense mutation in *STK11* (c.1062C > G) and three in *STK11IP* were identified.
• Whole genome sequencing could help predict cervical malignancy in patients with PJS/LEGH.

## Graphical abstract

**Figure:**
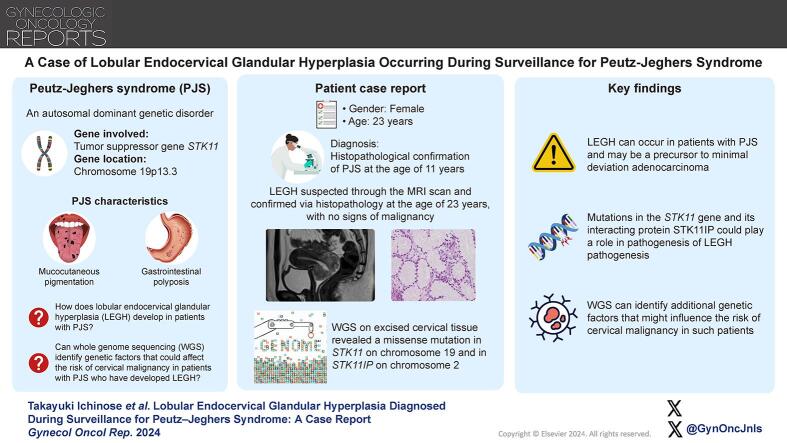

## 1 Introduction

Peutz–Jeghers syndrome (PJS) is an autosomal-dominant genetic disorder characterized by mucocutaneous pigmentation and gastrointestinal polyposis, excluding the esophagus. The incidence of this condition is estimated at 1 in 50,000 to 1 in 200,000 individuals. The tumor suppressor gene serine/threonine kinase 11 (*STK11*), located on chromosome 19p13.3, is responsible for PJS, with variants identified in approximately 80–94 % of cases (Hirasawa et al., 2012). Patients with PJS are predisposed to developing benign and malignant tumors in gastrointestinal and extra-gastrointestinal organs, including gynecological conditions such as sex-cord tumors with annular tubules and minimal deviation adenocarcinoma (MDA) (Kuragaki et al., 2003, Mikami et al., 2004). Additional mutations in *STK11* have recently been identified. These mutations affect key regulatory domains involved in kinase activity and cellular stress responses, thereby highlighting the crucial function of this gene in tumor suppression (Launonen et al., 2005). Lobular endocervical glandular hyperplasia (LEGH), considered an MDA precursor lesion, has been observed in patients with PJS, although its association with STK11 variants has not been well established. LEGH is a benign lesion that can mimic and potentially progress to well-differentiated gastric-type endocervical adenocarcinoma (GAS), including its aggressive subtype, MDA. This distinction is particularly challenging in HPV-independent tumors, where histological and immunophenotypic evaluation play crucial diagnostic roles. As highlighted by the previous publication (Molero et al., 2023), LEGH poses significant diagnostic challenges due to its overlapping features with GAS, necessitating careful histological evaluation, immunophenotyping, and molecular studies to confirm the diagnosis and rule out malignancy. Their findings emphasize the importance of integrating these diagnostic modalities to avoid misdiagnosis and ensure appropriate clinical management.

To our knowledge, genetic analysis has been performed in only one reported case. However, recent evidence indicates that alterations in *STK11*, particularly in its interaction with proteins such as STK11 interacting protein (STK11IP), may critically influence the development of LEGH and its progression to malignancy.

We present a case of LEGH that was diagnosed during a follow-up of a patient with PJS, the results of genetic testing, and the implications for the field.

## 2 Case report

**Fig. 1:**
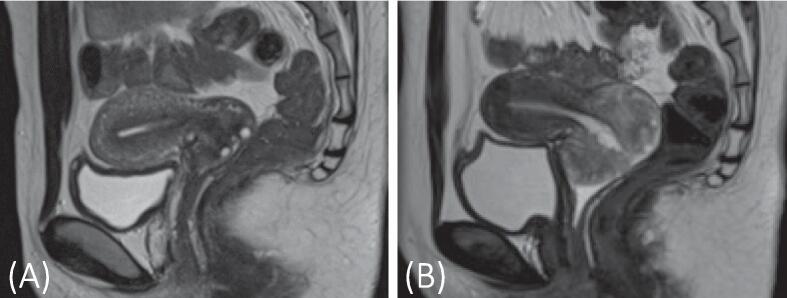
(A) T2-weighted MRI image from the initial gynecologic visit of the patient, indicating no suspicious findings in the cervix. (B) T2-weighted MRI image captured 17 months later, demonstrating a high signal intensity in the cervix, suggestive of LEGH. MRI, magnetic resonance imaging; LEGH, lobular endocervical glandular hyperplasia.

The patient, a 23-year-old woman, had hyperpigmentation on her fingertips since childhood and on her lips since nine years of age. At 11 years old, she was suspected of having PJS; upper gastrointestinal endoscopy revealed multiple polyps in the stomach, duodenum, and jejunum. Later that year, she underwent surgery for intussusception caused by small intestinal polyps, which involved resection of the gastric and jejunal polyps. Histopathology confirmed Peutz–Jeghers polyps. PJS was diagnosed based on the characteristic lip hyperpigmentation and histological findings. At 16 years old, she experienced another episode of intussusception and underwent another surgery for small intestinal polyps. A computed tomography scan performed for abdominal distention at 22 years of age incidentally detected a cervical lesion, leading to a gynecology referral. HPV testing was not performed. Cervical cytology indicated the presence of atypical glandular cells (AGC) and the possibility of LEGH or MDA. Pelvic magnetic resonance imaging (MRI) showed cystic lesions in the cervix but no typical which is called cosmos sign (Takatsu et al., 2011), or elevated tumor markers (Fig. 1A); thus, the patient was scheduled for follow-up after three months. However, she did not return until one year and five months later, complaining of increased vaginal discharge. Cervical cytology indicated AGC, with no tumor marker elevation. Transvaginal ultrasound revealed an enlarged cervix and pelvic contrast-enhanced MRI showed an enlarged cervical lesion, raising suspicion of MDA (Fig. 1B). A cervical conization was performed for diagnostic purposes.

**Fig. 2:**
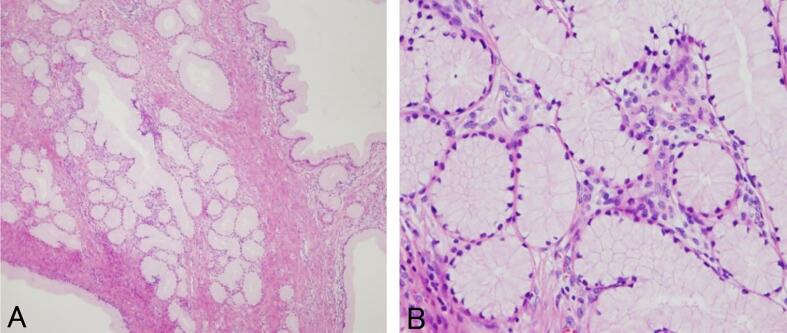
Histopathological examination of the cervical lesion. (A) Hematoxylin and eosin (HE)-stained tissue at 200 × magnification. (B) HE-stained tissue at 400 × magnification, highlighting morphological characteristics indicative of LEGH. LEGH, lobular endocervical glandular hyperplasia.

Histopathological examination confirmed LEGH with residual lesions on the endocervical side of the uterus (Fig. 2A and B). After obtaining informed consent, high-throughput next-generation sequencing (NGS) of the excised cervical tissue revealed a missense mutation in *STK11* on chromosome 19 (c.1062C > G) and three missense mutations in *STK11IP* on chromosome 2 (c.2G > T, c.1687G > A, c.2255C > T) (Table 1, Supplemental Material 1). No other pathologic variants were detected. Whole genome sequencing and bioinformatics analyses were performed at RIKEN GENESIS CO., LTD. Given the absence of atypical LEGH and MDA pathology in the resected specimen and the desire of the patient to preserve fertility, she was prescribed follow-up monitoring. Five years have passed since the surgery, and no malignancy has been observed.

**Table 1:** Mutations identified in the cervical lesion of the patient.

Chrom	Chrom Start	Chrom End	References	Alternatives	Type	Known/Novel	Gene	Function
chr19	1,223,125	1,223,125	C	G	SNV	Known	STK11	Missense
chr2	220,462,640	220,462,640	G	T	SNV	Known	STK11IP	Missense
chr2	220,473,355	220,473,355	G	A	SNV	Known	STK11IP	Missense
chr2	220,476,443	220,476,443	C	T	SNV	Known	STK11IP	Missense

Chrom (or chr), chromosome; SNV, single nucleotide variant; *STK11*, serine/threonine kinase 11; *STK11IP*, serine/threonine kinase 11 interacting protein.

This case suggests the involvement of PJS in the pathogenesis of LEGH and highlights the potential utility of whole genome sequencing in predicting cervical malignancy in patients with PJS. Recent insights into the molecular mechanisms of STK11IP suggest that its interaction with STK11 influences the progression of precursor lesions such as LEGH, making it a critical area of study for potential therapeutic targets (Pons-Tostivint et al., 2021).

## 3 Discussion

Lobular endocervical glandular hyperplasia (LEGH), first proposed by Nucci et al. (1999), is a benign pseudoneoplastic lesion of the cervix that must be differentiated from Minimal deviation adenocarcinoma (MDA). LEGH expresses gastric mucin and histologically exhibits pyloric gland-type features, serving as a precursor lesion to MDA (Ito et al., 2012). The coexistence of LEGH with atypical LEGH or MDA has been reported in Peutz–Jeghers syndrome (PJS), suggesting that LEGH represents an early stage in the development of MDA (Nishio et al., 2009).

Certain mutations in *STK11*, particularly those affecting its regulatory domains, significantly impact its tumor-suppressive functions (Kinzler and Vogelstein, 1996, Sanchez-Cespedes, 2007). These mutations may accelerate the progression of precursor lesions, such as LEGH, into more malignant forms, such as MDA, supporting the hypothesis that LEGH may be a precursor to MDA, with *STK11* influencing this progression (Kuragaki et al., 2003; Takatsu et al., 2013).

Beyond the cervix, pyloric gland metaplasia and differentiation have been observed in the jejunum, bladder, ovary, and fallopian tubes in patients with PJS, indicating that pyloric gland transformation is a unique event associated with PJS. In the case of PJS with a LEGH-like ovarian tumor, a heterozygous deletion mutation in *STK11* was identified, further supporting the significance of *STK11* variants in the proliferation of pyloric gland-type mucous epithelia across various anatomical sites (Chiraphapphaiboon et al., 2023).

The protein STK11IP, which interacts with STK11, plays a key role in modulating STK11 activity, particularly during cellular stress, potentially influencing the pathogenesis of LEGH and related lesions. Targeting the STK11/STK11IP axis could represent a therapeutic strategy (Shackelford and Shaw, 2009). To the best of our knowledge, only four cases of LEGH in PJS have been reported to date, and STK11 variants were investigated in only one of these cases. In the present case, NGS revealed a missense mutation in *STK11* on chromosome 19 (c.1062C > G) and in *STK11IP* on chromosome 2 (c.2G > T, c.1687G > A, c.2255C > T), indicating that STK11 variants in PJS may contribute to LEGH pathogenesis. *STK11IP*, consisting of 25 exons and mapped to chromosome 2q36, encodes a 121 kDa protein that interacts with STK11 to negatively regulate transforming growth factor β signaling. However, no pathologic mutations in *STK11IP* have been conclusively linked to PJS, and further research is required to clarify its role. The newly discovered regulatory functions of STK11IP may provide new avenues for understanding and treating lesions and tumors in patients with PJS (Shackelford and Shaw, 2009).

PJS is associated with a high risk of malignancy, with a *meta*-analysis indicating that 186 out of 583 Japanese patients developed malignancies, a significant proportion of which were gynecologic cancers. In a *meta*-analysis of 583 Japanese PJS patients, 31.9 % developed malignancies, with gynecologic cancers accounting for a significant proportion. Among these, uterine malignancies, including minimal deviation adenocarcinoma (MDA), were predominant. The risk of gynecologic malignancies was found to increase with age, with a 14.6 % risk at 30 years, 29.2 % at 40 years, 49 % at 50 years, and 55.4 % by 60–70 years. Furthermore, the incidence of MDA among women with malignancies was 46.8 % (52/111), underscoring its importance in this population. (Iwamuro, 2019). The median age of onset for uterine cancer (including MDA) was 32.5 years, highlighting the need for early surveillance. Variants in *STK11* exons have been associated with differing cancer risks. For example, mutations in exon 1 may be more commonly linked to gastrointestinal cancers, whereas exon 8 mutations have shown a stronger correlation with gynecologic malignancies, including MDA. However, the overall impact of specific exon mutations on cancer development remains uncertain and varies across studies. The identification of a somatic *STK11* mutation via tumor WES highlights its potential role in tumorigenesis but does not imply germline predisposition or hereditary risk. Early genetic screening, which applies to germline testing, is not applicable in this case. The recent findings regarding STK11 and STK11IP suggest that the interaction of these proteins could be crucial for preventing malignancy in patients with PJS, highlighting the importance of early genetic screening and monitoring (Gurumurthy et al., 2001). More research is needed to determine how somatic mutations contribute to malignancy progression and whether they hold therapeutic or prognostic significance.

This case underscores the potential utility of whole-genome sequencing in patients with PJS and LEGH, particularly for those desiring fertility preservation. Genetic analyses can provide insights into molecular changes associated with cervical malignancy, aiding in more nuanced clinical decision-making and potentially avoiding unnecessary hysterectomies in select cases. While the findings suggest a role for genetic testing in understanding cancer risk, the use of companion diagnostic testing as a routine predictor of malignancy risk remains premature. Further studies are needed to aggregate genetic data and clarify the relationship between *STK11* variants and cancer risk in PJS. Such research could ultimately contribute to the development of personalized treatment and surveillance strategies in this patient population.

## CRediT authorship contribution statement

**Takayuki Ichinose:** Writing – review & editing, Writing – original draft, Data curation, Conceptualization. **Kazuki Takasaki:** Writing – review & editing, Writing – original draft, Data curation, Conceptualization. **Yuko Takahashi:** Writing – review & editing, Data curation. **Mana Hirano:** Writing – review & editing, Data curation. **Haruka Nishida:** Writing – review & editing, Data curation. **Haruko Hiraike:** Writing – review & editing, Data curation. **Yuko Sasajima:** Writing – review & editing, Data curation. **Kazunori Nagasaka:** Writing – review & editing, Writing – original draft, Supervision, Data curation, Conceptualization.

## Funding

This work was supported by a Grant-in-Aid for Scientific Research C (grant no. 22 K09554 to K.N.) from the Ministry of Education, Science, and Culture, Japan.

## Declaration of competing interest

The authors declare that they have no known competing financial interests or personal relationships that could have appeared to influence the work reported in this paper.

## Data Availability

Data will be made available on request.
